# The burden of oral cancer in China, 1990–2017: an analysis for the Global Burden of Disease, Injuries, and Risk Factors Study 2017

**DOI:** 10.1186/s12903-020-01386-y

**Published:** 2021-01-28

**Authors:** Yang Yang, Maigeng Zhou, Xinying Zeng, Chunxiao Wang

**Affiliations:** 1 National Center for Chronic and Noncommunicable Disease Control and Prevention, Chinese Center for Disease Control and Prevention, No. 27 Nanwei Road, Xicheng District, Beijing, 100050 China; 2 Chinese Center for Disease Control and Prevention, No. 27 Nanwei Road, Xicheng District, Beijing, 100050 China

**Keywords:** Oral cancer, Incidence, Mortality, DALY, China, Disease burden

## Abstract

**Background:**

Oral cancer is among the most common malignant tumors worldwide, and it has become an increasingly important public health problem in China. This study systematically assesses the current state of oral cancer in China from 1990 to 2017, providing new information and perspectives for oral health researchers and public health policy makers.

**Methods:**

Based on the Global Burden of Disease, Injuries, and Risk Factors Study 2017 (GBD 2017), we evaluated the incidence rates, mortality and disability-adjusted life year (DALY) rates for oral cancer in China and their changing trends between 1990 and 2017, making comparisons by gender and age. We also assessed the DALY rates associated with oral cancer at the provincial level for 33 provinces and their trends over time.

**Results:**

From 1990 to 2017, the number of new cases and the age-standardized incidence rate for oral cancer in China increased by 280.0% and 79.7%, respectively; the number of deaths and the age-standardized mortality rose by 196.8% and 29.0%, respectively; and the number of DALYs and the age-standardized DALY rate increased by 149.1% and 21.0%, respectively. The incidence rates for oral cancer rose after 30 years of age and peaked at 65–69 years; the mortality for oral cancer rose after 50 years of age and peaked at 65–69 years; and the DALY rates for oral cancer rose after 45 years of age and peaked at 65–69 years. The incidence rates, mortality and DALY rates for oral cancer in males were significantly higher than those in females and showed an upward trend, while there was a decrease or no significant change in females. The DALY rates increased in 21 provinces and decreased in 12 provinces, with the largest growth in Henan Province and the largest decline in Hong Kong Province.

**Conclusions:**

The burden of oral cancer in China continues to increase continuously. More prevention, control and intervention measures should be taken and increased attention paid to common risk factors is essential for the prevention of oral cancer.

## Background

Oral cancer, a subset of cancer, is often ignored by the government in public health policy, but it has become an important component of global oral health problems, with a significant economic impact on society and the economy [1]. The major site of oral cancer in both Asian and non-Asian patients is the tongue, followed by the oral buccal mucosa and gums in Asian regions, and the floor of the mouth, lip and alveolar mucosa in non-Asian regions [2]. The site of oral cancer depends on the main risk factors for the particular geographical location [3, 4]. The major frequently found pathological classification of oral cancer is oral squamous cell carcinoma, which accounts for up to 90% of all oral malignant tumours [5]. Despite improvements in early diagnosis and early treatment techniques, the 5-year survival rate is still very low for oral cancer, ranging from 40 to 50% [6].

Global Cancer Statistics 2018, compiled by the International Agency for Research on Cancer (IARC), provides a comprehensive report on the current status of cancer around the world [7]. This recent report estimates that oral cancer accounted for almost 2.0% of all cancer cases and 1.9% of all cancer deaths globally [7].

According to GLOBOCAN 2018 estimates, the incidence rates for oral cancer varied across different geographic areas. The highest incidence rates for oral cancer were in Melanesia, followed by South Central Asia, with age-standardized incidence rates of more than 12 per 100,000. In contrast, the lowest incidence rates for oral cancer were in Central America and West Africa, which were less than 3 per 100,000 population [7].

Oral cancer tended to be concentrated in certain high-risk areas [4]. It is very prevalent in South Asia (especially India and Sri Lanka) and the Pacific Islands, with Papua New Guinea having high incidence rates worldwide in both males and females [7]. Oral cancer was also a major cause of death among males in India and Sri Lanka. It is worth noting that the incidence and mortality of oral cancer are more serious in developing countries, as they are usually two to three times higher than those in developed territories and countries [1, 8]. Because of the heavy socioeconomic burden, oral cancer should receive greater attention in developing countries.

The cancer statistics for the incidence and mortality in China usually combine oral cancer and oropharyngeal cancer. The Cancer Registry Report, released by the National Central Cancer Registry of China (NCCR), summarizes the national cancer registration information. The latest report published in 2020 estimates that in 2015, there were about 4.29 million new cancer cases and 2.81 million cancer deaths in China, while there were about 48,100 new cases and 22,100 deaths from oral and oropharyngeal cancer, accounting for 1.12% and 0.79% of all new cancer cases and deaths, respectively [9, 10]. Although the absolute numbers are not high, the burden of oral and oropharyngeal cancer has been on the rise in China in recent years.

The main risk factors for oral cancer in China are smoking and alcohol use. In particular, it is important to note that betel quid chewing is also an important risk factor in China [11]. In 2018, the number of smokers in China was 308 million, 50.5% of whom were male and only 2.1% of whom were female [12]. The incidence of oral cancer in Chinese males is also much higher than that in females. The drinking rate among adults in China is 30.5%, including 53.8% for men and 12.2% for women [13]. In China, betel quid chewing habits are mainly distributed in Hunan, Hainan, and Yunnan provinces, especially Hunan Province. It has been reported that betel quid chewing is common in Hunan Province, and the proportion of betel nut chewers is as high as 64.5–82.7% [14].

At present, comprehensive data on the burden of oral cancer are lacking at the national and provincial levels. Therefore, an analysis of the change trend in oral cancer in China over the last three decades fills a significant gap and can help to inform oral health researchers and public health policy makers in China to focus more attention on oral cancer. In this study, we showed the incidence rates, mortality, and DALY rates for oral cancer in China from 1990 to 2017.

## Methods

### Data sources

The data for this study were obtained from the 2017 Global Burden of Disease, Injuries and Risk Factor study. The GBD 2017 includes cause-specific mortality from 282 causes of deaths in 195 regions and countries around the world between 1990 and 2017, as well as health losses from 359 diseases and injuries associated with DALYs and 84 risk factors, addressing a total of 23 age groups and two genders [15–22].

The provincial administrative units of this study include twenty-two provinces, five autonomous regions, four municipalities directly under the Central Government in the Chinese Mainland, and the Hong Kong and Macao Special Administrative regions. The above 33 locations are collectively termed provinces throughout the whole article.

These data mainly come from surveys and surveillance. The primary data sources for Chinese mainland included the China Disease Surveillance Point (DSP) System, the China Cancer Registry data, and the vital registration gathered by the Chinese Center for Disease Control and Prevention (CDC), supplement by hospital inpatient data, published literature, and some national-level surveys [16, 18]. The National Census and the Maternal and Child Surveillance System were also important complements to the data sources. In addition, the cause of death data for Hong Kong and Macao come from medical data records certified by the World Health Organization (WHO) [17]. This study is not included in the data of Taiwan Province because it is not available.

### Eligibility criteria

The aetiological code used in GBD 2017 was based on the International Classification of Disease (ICD) formulated by the WHO, which is the current standard in the world and the most exhaustive cause list [23]. The definition of oral cancer in GBD 2017 consisted of codes C00-C08 (lip and oral cavity cancer) in ICD10. Codes C11 (nasopharyngeal cancer), C09–C10 and C12–C13 (cancer of other parts of the pharynx), and C14 (malignant neoplasm of other and ill-defined sites in the lip, oral cavity, and pharynx) were excluded by GBD 2017 [15]. Specific coding by oral cancer type can be found in full detail in the GBD 2017 literature [17].

### Incidence, mortality and DALYs

Details of the methods used in GBD 2017 have been fully described elsewhere [14–22]. In this study, the disease burden of oral cancer is described by incidence, mortality and DALYs in China. To comprehensively measure and compare the heath loss from fatal and non-fatal disease burden, a summary indicator is created called DALY to estimate trends in population health over the past 27 years, and it is composed of years lived with disability (YLDs) and years of life lost (YLLs) [20]. YLDs is an indicator measuring the sequelae of the disease, and it is computed by multiplying the prevalence by the disability weight for the health associated with the sequela of the disease. YLLs is an indicator of health loss used to assess premature death, and it is the result of multiplying the number of deaths from various causes and the standard life expectancy per year.

### Possible methods

The GBD Cause of Death Ensemble modelling tool, CODEm, developed by GBD, was used to estimate cause-specific mortality for each location, year, age, and gender. The estimates of nonfatal disease burden, such as incidence, were commonly used with the Bayesian meta-regression tool DisMod-MR 2.1. The age-standardized population of GBD 2017 was calculated using the age of the world population as the standard [21]. Our research followed the principles in the Guidelines for Accurate and Transparent Health Estimates Reporting (GATHER) to obtain transparent as well as replicable data and results [24]. All data and results can be obtained from the Global Health Data exchange (GHDx) on the GBD website [15, 20].A comprehensive list of core data was provided by the GHDx source tools (http://ghdx.healthdata.org/data-type/disease-registry), such as data on deaths, YLLs, YLDs, DALYs, prevalence, incidence, causes, risks, cause-risk attribution, aetiologies, and impairment.

### Statistical analyses

Analyses were done using Python versions 2.7.12 and 2.7.3, Stata version 13.1, and R version 3.2.2. The incidence of cancer was estimated directly from cancer mortality using the mortality-to-incidence ratios (MIRs). MIR estimation and estimation of cancer mortality have been explained in detail elsewhere. The incidence and mortality data from the cancer registrations were matched according to cancer, age, sex, year and location to produce MIRs. MIRs were estimated using a fixed effect logistic regression model, with covariates for sex, categorical age, and the Healthcare Access and Quality index [15].

We generated 1000 draws from each step of the evaluation process. Then, 95% uncertainty intervals (UIs) were computed using the 2.5th and 97.5th percentile of the ordered 1000 estimate values, and point estimates were calculated by the mean values across the 1000 draws. Unlike confidence intervals, which derived uncertainty only from sampling errors, UIs acquire uncertainty from multiple modelling steps, model estimation and model specification [15].

## Results

### Overall trends

From 1990 to 2017, the total number of cases of oral cancer in China increased by 289.2% from 49.3 thousand (95% UI: 47.0 thousand–51.7 thousand) to 192.1 thousand (95% UI: 180.9 thousand–201.8 thousand), and the number of new cases of oral cancer in China increased by 280.0%, from 12.4 thousand (95% UI: 11.8 thousand–13.0 thousand) to 47.2 thousand (95% UI: 44.5 thousand–49.7 thousand). It was estimated that the age-standardized incidence rate per 100,000 rose by 79.7% from 1.36 (95% UI: 1.29–1.43) in 1990 to 2.44 (95% UI: 2.30–2.57) in 2017 (Table 1). Compared with 1990 values, the total number of deaths from oral cancer in 2017 increased by 196.8%, from 6.9 thousand (95% UI: 6.6 thousand–7.4 thousand) to 20.7 thousand (95% UI: 19.6 thousand–21.8 thousand), respectively, and the age-standardized mortality per 100,000 rose by 29.0% from 0.84 (95% UI: 0.80–0.88) in 1990 to 1.09 (95% UI: 1.03–1.14) in 2017 (Table 2). From 1990 to 2017, the number of DALYs from oral cancer increased by 149.1% from 204.6 thousand (95% UI: 194.1 thousand–215.2 thousand) to 509.7 thousand (95% UI: 478.2 thousand–538.1 thousand), respectively, and the age-standardized DALY rate per 100,000 rose by 21.0% from 21.0 (95% UI: 19.9–22.1) to 25.4 (95% UI: 23.8–26.8) (Table 3).

**Table 1:** Number of cases and new cases and age-standardized incidence rates for oral cancer in China, 1990–2017

	Cases (n)			New cases (n)			Incidence rate per 100,000		
	1990	2017	Change (%)	1990	2017	Change (%)	1990	2017	Change (%)
*All*	49362.8 (47,023.4, 51,733.6)	192141.9 (180,958.9, 201, 822.5)	289.2 (284.8, 290.1)	12,429.6 (11,820.0, 13,045.5)	47,214.5 (44,501.9, 49,658.0)	280.0 (276.4, 281.0)	1.36 (1.29, 1.43)	2.44 (2.30, 2.57)	79.7 (78.0, 79.9)
*Ages*									
1–4 years	0.0 (0.0, 0.0)	0.0 (0.0, 0.0)	–	0.0 (0.0, 0.0)	0.0 (0.0, 0.0)	–	0.00 (0.00.0.00)	0.00 (0.00.0.00)	–
5–9 years	0.0 (0.0, 0.0)	0.0 (0.0, 0.0)	–	0.0 (0.0, 0.0)	0.0 (0.0, 0.0)	–	0.00 (0.00.0.00)	0.00 (0.00.0.00)	–
10–14 years	0.0 (0.0, 0.0)	0.0 (0.0, 0.0)	–	0.0 (0.0, 0.0)	0.0 (0.0, 0.0)	–	0.00 (0.00.0.00)	0.00 (0.00.0.00)	–
15–19 years	752.1 (685.3, 827.5)	458.6 (411.8, 509.6)	− 39.0 (− 39.9, − 38.4)	152.9 (139.5, 167.8)	75.3 (67.6, 83.9)	− 50.7 (− 51.6, − 50.0)	0.12 (0.11, 0.13)	0.10 (0.09, 0.11)	− 18.6 (− 20.1, − 17.5)
20–24 years	1604.3 (1464.2, 1756.9)	977.5 (862.8, 1095.2)	− 39.1 (− 41.4, − 37.7)	313.4 (287.1, 341.8)	158.1 (139.0, 177.9)	− 49.6 (− 51.6, − 48.0)	0.24 (0.22, 0.26)	0.17 (0.15, 0.20)	− 27.2 (− 30.1, − 24.9)
25–29 years	2654.6 (2434.4, 2903.9)	3344.6 (2991.8, 3746.2)	26.0 (22.9, 29.0)	511.0 (469.5, 556.6)	540.4 (482.5, 605.9)	5.8 (2.8, 8.9)	0.46 (0.43, 0.51)	0.44 (0.39, 0.49)	− 5.3 (− 7.9, − 2.5)
30–34 years	4048.6 (3728.3, 4406.6)	6739.9 (6090.2, 7469.9)	66.5 (63.4, 69.5)	811.8 (745.3, 884.3)	1109.3 (998.4, 1231.4)	36.6 (34.0, 39.3)	0.92 (0.84, 1.00)	0.93 (0.84.1.03)	1.2 (− 0.8, 3.1)
35–39 years	4396.5 (3997.1, 4746.9)	6774.3 (6030.5, 7531.9)	55.0 (50.9, 58.7)	899.1 (821.5, 979.0)	1124.7 (996.8, 1231.4)	25.1 (21.3, 28.3)	0.98 (0.90, 1.07)	1.16 (1.02, 1.29)	17.6 (14.1, 20.6)
40–44 years	3306.2 (3050.3, 3586.1)	7394.9 (6695.3, 8163.8)	123.7 (119.5, 127.7)	802.8 (739.3, 871.3)	1444.4 (1301.3, 1598.4)	79.9 (76.0, 83.5)	1.19 (1.10, 1.30)	1.30 (1.17, 1.44)	9.2 (6.8, 11.3)
45–49 years	3657.3 (3394.7, 3952.1)	14,896.5 (13,594.8, 16,254.4)	307.3 (300.5, 311.3)	911.9 (845.6, 986.2)	3196.2 (2898.5, 3493.5)	250.5 (242.8, 254.2)	1.76 (1.64, 1.91)	2.56 (2.32, 2.80)	45.2 (42.0, 46.7)
50–54 years	4202.0 (3896.8, 4534.8)	23,082.2 (21,084.5, 25,047.5)	449.3 (441.1, 452.3)	1055.8 (979.3, 1140.0)	5081.7 (4620.7, 5529.7)	381.3 (371.8, 385.0)	2.21 (2.05, 2.38)	4.25 (3.87, 4.63)	92.5 (88.7, 94.0)
55–59 years	5008.8 (4644.7,5431.0)	17,934.0 (16,658.1, 19,326.4)	258.1 (258.4, 255.9)	1271.5 (1178.3, 1379.3)	4160.5 (3845.6, 4496.7)	227.2 (226.0, 226.4)	2.93 (2.71, 3.17)	5.16 (4.77, 5.58)	76.5 (75.9, 76.1)
60–64 years	5303.4 (4924.7, 5722.7)	27,153.3 (25,178.2, 29,235.5)	412.0 (410.9, 411.3)	1376.0 (1276.4, 1484.4)	6692.7 (6194.3, 7220.6)	386.4 (385.3, 386.4)	3.88 (3.60, 4.19)	8.08 (7.48, 8.72)	108.3 (107.8, 108.3)
65–69 years	5123.4 (4735.1, 5515.7)	29,990.9 (27,757.7, 32, 361.9)	485.4 (486.0, 486.7)	1376.7 (1270.8, 1482.2)	7627.3 (7049.3, 8256.6)	454.0 ( 454.7, 457.1)	5.03 (4.64, 5.42)	12.46 (11.52, 13.49)	147.7 (148.0, 149.1)
70–74 years	4268.6 (3943.0, 4600.1)	21,902.2 (20,118.1, 23,825.7)	413.1 (410.2, 417.9)	1218.5 (1125.3, 1314.0)	5918.4 (5439.5, 6441.0)	385.7 (383.4, 390.2)	6.46 (5.97, 6.97)	14.52 (13.35, 15.80)	124.8 (123.7, 126.8)
75–79 years	2888.5 (2685.9, 3110.2)	16,714.5 (15,479.2, 18,029.2)	478.7 (476.3, 479.7)	897.7 (834.1, 967.9)	4861.9 (4496.1, 5246.4)	441.6 (439.0, 442.0)	7.86 (7.30, 8.47)	17.21 (15.92, 18.58)	119.0 (118.0, 119.2)
≥ 80 years	2175.4 (2036.4, 2319.1)	14,778.8 (13,865.1, 15,681.1)	579.4 (576.2, 580.9)	830.6 (779.6, 885.0)	5223.7 (4889.9, 5545.1)	528.9 (526.6, 527.2)	9.91 (9.31, 10.56)	17.71 (16.58, 18.80)	78.6 (78.0, 78.2)
*Gender*									
Male	25,816.0 (23,924.6, 28,059.8)	138,715.7 (127,853.3, 148,106.9)	437.3 (427.8, 434.4)	6912.7 (6411.7, 7510.5)	35,743.0 (32,980.3, 38,075.3)	417.1 (407.0, 414.4)	1.58 (1.46, 1.71)	3.79 (3.49, 4.03)	140.0 (135.3, 139.0)
Female	23,546.9 (22,287.4, 25,020.9)	53,426.3 (50,140.4, 56,730.7)	126.8 (125.0, 126.7)	5516.9 (5226.9, 5895.1)	11,471.5 (10,776.1, 12,151.9)	107.9 (106.1, 106.2)	1.17 (1.11, 1.26)	1.19 (1.12, 1.26)	1.1 (− 0.4, 0.3)

**Table 2:** Number of deaths and age-standardized mortality for oral cancer in China, 1990–2017

	Deaths (n)			Mortality per 100,000		
	1990	2017	Change (%)	1990	2017	Change (%)
*All*	6995.3 (6641.9, 7350.1)	20,760.8 (19,614.4, 21,816.8)	196.8 (195.3, 196.8)	0.84 (0.80, 0.88)	1.09 (1.03, 1.14)	29.0 (28.4, 29.0)
*Ages*						
1–4 years	0.0 (0.0, 0.0)	0.0 (0.0, 0.0)	–	0.0 (0.0, 0.0)	0.0 (0.0, 0.0)	–
5–9 years	0.0 (0.0, 0.0)	0.0 (0.0, 0.0)	–	0.0 (0.0, 0.0)	0.0 (0.0, 0.0)	–
10–14 years	0.0 (0.0, 0.0)	0.0 (0.0, 0.0)	–	0.0 (0.0, 0.0)	0.0 (0.0, 0.0)	–
15–19 years	47.0 (43.4, 50.8)	13.4 (12.2, 14.8)	− 71.5 (− 72.0, − 70.9)	0.04 (0.03, 0.04)	0.02 (0.02, 0.02)	− 53.0 (− 53.8, − 52.0)
20–24 years	84.6 (78.4, 91.1)	24.5 (21.6, 27.4)	− 71.0 (− 72.4, − 69.9)	0.06 (0.06, 0.07)	0.03 (0.02, 0.03)	− 58.2 (− 60.2, − 56.6)
25–29 years	120.1 (111.7, 129.5)	73.8 (66.7, 81.7)	− 38.5 (− 40.3, − 36.9)	0.11 (0.10, 0.12)	0.06 (0.05, 0.07)	− 44.9 (− 46.5, − 43.5)
30–34 years	211.6 (195.7, 228.8)	169.5 (154.2, 185.7)	− 19.9 (− 21.2, − 18.8)	0.24 (0.22, 0.26)	0.14 (0.13, 0.16)	− 40.7 (− 41.7, − 39.9)
35–39 years	271.8 (251.9, 293.8)	198.2 (176.7, 221.5)	− 27.1 (− 29.9, − 24.6)	0.30 (0.28, 0.32)	0.20 (0.18, 0.23)	− 31.4 (− 34.0, − 29.1)
40–44 years	376.8 (346.2, 408.3)	441.4 (399.9, 483.7)	17.1 (15.5, 18.4)	0.56 (0.51, 0.61)	0.40 (0.36, 0.44)	− 28.9 (− 29.9, − 28.1)
45–49 years	488.3 (454.3, 527.0)	1170.6 (1070.8, 1271.0)	139.7 (135.7, 141.2)	0.94 (0.88, 1.02)	0.94 (0.86, 1.02)	− 0.7 (− 2.4, − 0.1)
50–54 years	585.0 (542.5, 632.0)	1940.1 (1789.8, 2092.8)	231.6 (229.9, 231.1)	1.22 (1.13, 1.32)	1.62 (1.50, 1.75)	32.7 (32.0, 32.4)
55–59 years	756.5 (702.3, 817.0)	1730.9 (1608.4, 1848.9)	128.8 (126.3, 129.0)	1.74 (1.62, 1.88)	2.15 (2.00, 2.30)	23.4 (22.1, 23.6)
60–64 years	879.3 (817.6, 949.2)	3004.6 (2805.8, 3222.5)	241.7 (239.5, 243.2)	2.48 (2.31, 2.68)	3.63 (3.39, 3.89)	46.3 (45.4, 46.9)
65–69 years	894.5 (829.1, 958.8)	3349.9 (3132.7, 3588.8)	274.5 (274.3, 277.8)	3.27 (3.03, 3.50)	5.47 (5.12, 5.86)	67.5 (67.4, 68.9)
70–74 years	827.4 (767.2, 887.3)	2692.5 (2495.0, 2876.3)	225.4 (224.2, 225.2)	4.39 (4.07, 4.70)	6.61 (6.12, 7.06)	50.6 (50.0, 50.5)
75–79 years	678.6 (632.6, 728.4)	2485.3 (2338.0, 2639.7)	266.2 (262.4, 269.6)	5.94 (5.54, 6.38)	8.80 (8.28, 9.35)	48.1 (46.6, 49.5)
≥ 80 years	773.7 (728.5, 821.1)	3466.1 (3295.2, 3620.8)	348.0 (341.0, 352.3)	9.23 (8.70, 9.80)	11.75 (11.17, 12.28)	27.3 (25.3, 28.5)
*Gender*						
Male	4140.3 (3837.3, 4483.2)	16,057.8 (14,918.5, 17,094.0)	287.8 (281.3, 288.8)	1.03 (0.96, 1.12)	1.73 (1.61, 1.84)	67.8 (64.7, 68.6)
Female	2854.9 (2709.5, 3087.6)	4703.0 (4425.1, 4968.5)	64.7 (60.9, 63.3)	0.67 (0.64, 0.73)	0.49 (0.46, 0.51)	− 27.5 (− 29.8, − 28.0)

**Table 3:** Number of DALYs and age-standardized DALY rates for oral cancer in China, 1990–2017

	DALYs (n)			DALY rate per 100,000		
	1990	2017	Change (%)	1990	2017	Change (%)
*All*	204,612.6 (194,172.9, 215,294.4)	509,746.1 (478,229.8, 538,113.9)	149.1 (146.3, 149.9)	21.0 (19.9, 22.1)	25.4 (23.8, 26.8)	21.0 (19.6, 21.3)
*Ages*						
1–4 years	0.0 (0.0, 0.0)	0.0 (0.0, 0.0)	–	0.0 (0.0, 0.0)	0.0 (0.0, 0.0)	–
5–9 years	0.0 (0.0, 0.0)	0.0 (0.0, 0.0)	–	0.0 (0.0, 0.0)	0.0 (0.0, 0.0)	–
10–14 years	0.0 (0.0, 0.0)	0.0 (0.0, 0.0)	–	0.0 (0.0, 0.0)	0.0 (0.0, 0.0)	–
15–19 years	3381.4 (3125.2, 3658.0)	985.1 (891.7, 1089.9)	− 70.9 (− 71.5, − 70.2)	2.7 (2.5, 2.9)	1.3 (1.2, 1.4)	− 51.9 (− 52.0, − 51.7)
20–24 years	5687.7 (5270.3, 6132.1)	1688.5 (1493.6, 1899.9)	− 70.3 (− 71.7, − 69.0)	4.3 (4.0, 4.6)	1.8 (1.6, 2.1)	− 57.2 (− 59.1, − 55.3)
25–29 years	7513.2 (6973.7, 8083.9)	4754.4 (4296.6, 5281.3)	− 36.7 (− 38.4, − 34.7)	6.8 (6.3, 7.3)	3.9 (3.5, 4.3)	− 43.3 (− 44.8, − 41.5)
30–34 years	12,164.7 (11,232.1, 13,133.1)	10,028.9 (9125.7, 10,991.5)	− 17.6 (− 18.8, − 16.3)	13.8 (12.7, 14.9)	8.4 (7.6, 9.2)	− 39.0 (− 39.8, − 38.0)
35–39 years	14,199.3 (13,148.9, 15,322.5)	10,646.7 (9477.7, 11,880.7)	− 25.0 (− 27.9, − 22.5)	15.5 (14.4, 16.7)	10.9 (9.7, 12.2)	− 29.5 (− 32.2, − 27.1)
40–44 years	17,594.9 (16,162.7, 19,066.8)	20,876.3 (18,879.2, 22,902.8)	18.6 (16.8, 20.1)	26.2 (24.0, 28.4)	18.8 (17.0, 20.7)	− 28.0 (− 29.1, − 27.1)
45–49 years	20,397.0 (18,976.9, 22,017.7)	49,313.4 (45,088.1, 53,673.8)	141.8 (137.6, 143.8)	39.4 (36.7, 42.6)	39.5 (36.1, 43.0)	0.1 (− 1.6, 1.0)
50–54 years	21,648.2 (20,107.3, 23,359.5)	72,651.9 (66,971.3, 78,474.1)	235.6 (233.1, 235.9)	45.3 (42.1, 48.9)	60.8 (56.0, 65.7)	34.3 (33.2, 34.4)
55–59 years	24,342.5 (22,573.0, 26, 310.3)	56,264.2 (52,196.2, 60,227.6)	131.1 (128.9, 131.2)	56.0 (51.9, 60.5)	69.8 (64.8, 74.8)	24.7 (23.5, 24.7)
60–64 years	24,206.8 (22,516.7, 26,156.4)	83,408.0 (77,764.8, 89,569.2)	244.6 (242.4, 245.4)	68.3 (63.5, 73.8)	100.8 (93.9, 108.2)	47.5 (46.6, 47.9)
65–69 years	20,587.9 (19,084.8, 22,084.0)	78,313.2 (73,105.8, 83,751.1)	280.4 (279.2, 283.1)	75.2 (69.7, 80.7)	127.9 (119.4, 136.8)	70.1 (69.9, 71.3)
70–74 years	15,502.2 (14,363.1, 16,644.6)	51,205.3 (47,493.9, 54,788.0)	230.3 (229.2, 230.7)	82.2 (76.1, 88.2)	125.6 (116.5, 134.4)	52.9 (52.3, 53.0)
75–79 years	9944.8 (9249.1, 10,666.1)	36,828.5 (34,508.5, 39,110.4)	270.3 (266.7, 273.1)	87.1 (81.0, 93.4)	130.4 (122.2, 138.5)	49.8 (48.3, 50.9)
≥ 80 years	7442.0 (6960.9, 7945.2)	32,781.7 (31,047.6, 34,399.2)	340.5 (333.0, 346.0)	88.8 (83.1, 94.8)	111.1 (105.3, 116.6)	25.1 (23.0, 26.7)
*Gender*						
Male	122,081.5 (113,302.5, 132,196.8)	400,756.2 (370,337.7, 428,177.2)	228.3 (223.9, 226.9)	25.2 (23.4, 27.3)	40.2 (37.1, 43.0)	59.7 (57.4.58.8)
Female	82,531.1 (78,339.8, 87,844.2)	108,989.9 (102,506.9, 115,591.5)	32.1 (30.8, 31.6)	16.9 (16.0, 18.1)	11.0 (10.3, 11.7)	− 35.0 (− 35.5, − 35.5)

### Age-specific trends

**Fig. 1:**
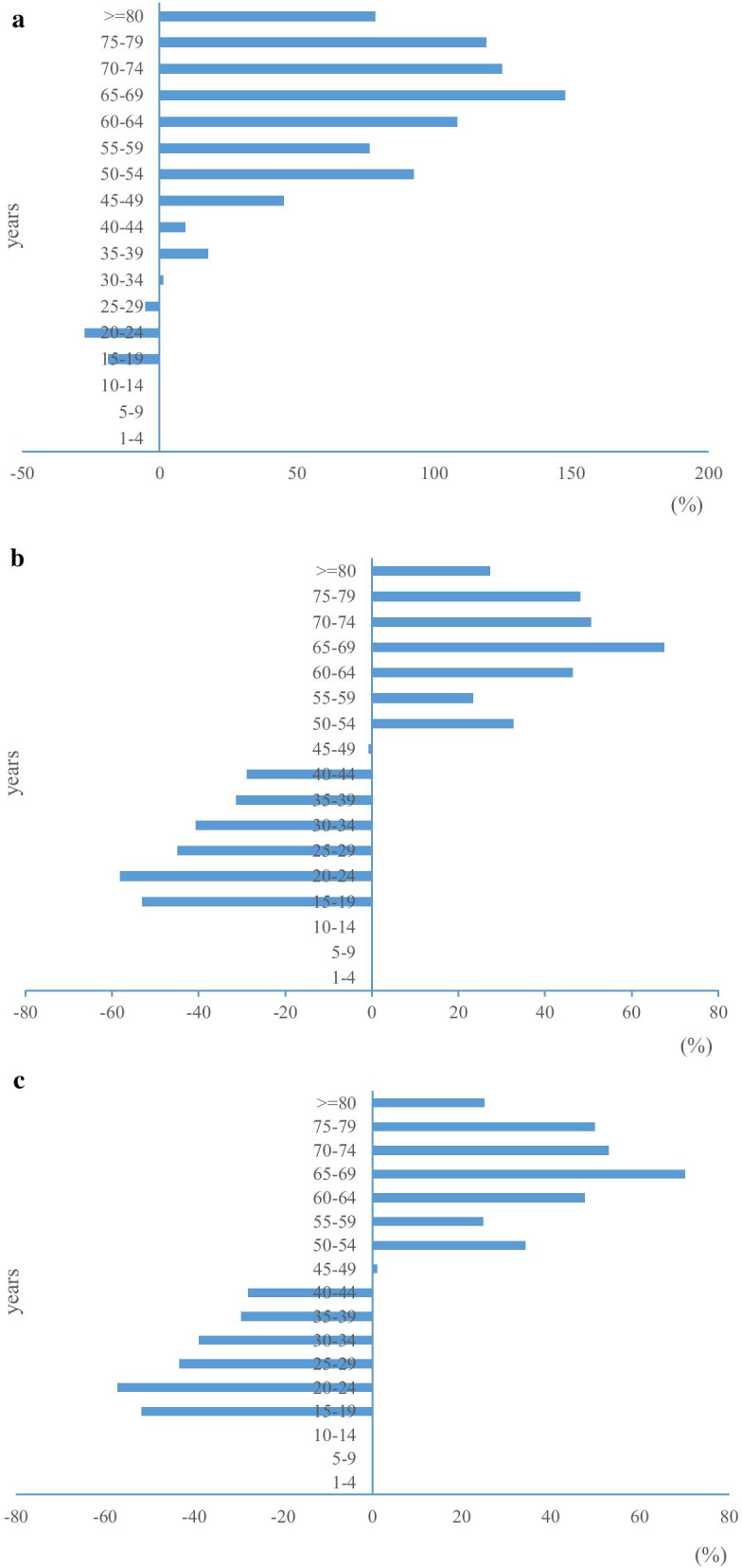
Changes of incidence, mortality, and DALY rates for oral cancer by age in China, 1990–2017. **a** Changes of incidence rates from 1990 to 2017 by age. **b** Changes of mortality from 1990 to 2017 by age. **c** Changes of DALY rates from 1990 to 2017 by age

We estimated the incidence rates, mortality and DALY rates per 100,000 population for all ages. Between 1990 and 2017, the incidence rates for oral cancer declined among those aged 29 years or younger and rose for those aged 30 years or older, peaking among those aged 65–69 years (Fig. 1a). During the same period, the mortality for oral cancer declined among those aged 49 years or younger and rose for those aged 50 years or older, peaking among those aged 65–69 years (Fig. 1b). During the same period, the DALY rates for oral cancer declined for those aged 44 years and younger and rose among those aged 45 years and older, peaking among those aged 65–69 years (Fig. 1c).

### Gender-specific trends

In 1990, the age-standardized incidence rate, mortality and DALY rate per 100,000 for males were 1.58, 1.03 and 25.2, respectively, while those for females were 1.17, 0.67 and 16.9. The values in males were higher than those in females in 1990. In 2017, the age-standardized incidence rate, mortality and DALY rate per 100,000 in males were 3.79, 1.73 and 40.2, respectively, and those in females were 1.19, 0.49 and 11.0. The values were higher for males than for females in 2017 (Tables 1, 2, and 3).

Comparing the changes between 1990 and 2017, it was found that the age-standardized incidence rate, mortality and DALY rate in males increased by 140.0%, 67.8% and 59.7%, respectively, while for females, the incidence rate increased by only 1.1%, and the mortality and DALY rate decreased by 27.5% and 35.0%, respectively (Tables 1, 2, and 3).

### Location-specific trends of DALYs

We estimated the age-standardized DALY rates and their changes for 33 provinces (Table 4). Table 4 shows the provincial age-standardized DALY rates per 100,000 and the changing trend in DALY rates between 1990 and 2017. In 1990, the age-standardized DALY rates of oral cancer were lowest in Shaanxi Province (15.2 per 100,000) and highest in Hong Kong Province (56.2 per 100,000). In 2017, the age-standardized DALY rates for oral cancer varied from a low of 13.0 per 100,000 in Ningxia Province to a high of 40.8 per 100,000 in Hunan Province.

**Table 4:** Age-standardized DALY rates and percentage changes by province, 1990–2017

Province	Age-standardized DALY rate per 100,000		Change (%)
	1990	2017	
Hong Kong	56.2	22.3	− 60.4
Beijing	19.5	13.6	− 30.2
Shanghai	20.1	14.4	− 28.5
Macao	19.8	14.2	− 28.3
Zhejiang	19.5	14.4	− 26.3
Ningxia	17.1	13.0	− 23.6
Tianjin	20.0	16.9	− 15.8
Anhui	25.1	21.6	− 13.9
Hainan	21.5	18.9	− 11.9
Tibet	16.9	16.6	− 2.1
Heilongjiang	19.5	19.1	− 2.1
Chongqing	20.3	20.1	− 1.0
Jiangsu	18.3	18.4	0.1
Xinjiang	20.0	20.0	0.1
Shandong	17.5	18.0	3.0
Jiangxi	19.9	21.9	10.1
Guangdong	23.0	25.7	12.0
Fujian	20.6	23.1	12.4
Hubei	18.6	21.8	17.4
Shanxi	22.5	26.7	18.7
Gansu	21.7	26.0	20.0
Qinghai	19.7	23.8	20.9
Jilin	30.8	37.6	21.9
Liaoning	22.0	28.1	28.0
Guangxi	20.3	26.1	28.3
Guizhou	29.2	38.7	32.6
Inner Mongolia	24.5	36.3	48.2
Yunnan	20.5	31.5	53.8
Hebei	23.5	36.1	53.9
Hunan	26.1	40.8	56.1
Shaanxi	15.2	24.7	62.6
Sichuan	20.1	33.7	67.2
Henan	15.5	26.2	68.4

Comparing the changes in the age-standardized DALY rate of oral cancer between 1990 and 2017, we can see that the age-standardized DALY rates decreased in 12 provinces, the largest decrease of which was 60.4% in Hong Kong Province, and increased in the other 21 provinces, the largest increase of which was 68.4% in Henan Province.

## Discussion

This study comprehensively and systematically assessed the burden of oral cancer in China between 1990 and 2017. The age-standardized DALY rate for oral cancer in China in 2017 was 25.4 per 100,000, corresponding to an increase of 21.0% over the 1990 rate. Over the same period, the age-standardized DALY rates for oral cancer worldwide were 60.6 and 64.2 per 100,000, respectively, representing an increase of 0.6% [20]. A Sub-Saharan African region research showed that the age-standardized DALY rates for oral cancer in this region were 47.8 and 45.0 per 100,000 between 1990 and 2017, respectively, with a decrease of 4.6% [25]. Although the age-standardized DALY rates for oral cancer in China were lower than those in the Sub-Saharan African region and below the worldwide average, the changing trend in the DALY rates for oral cancer in China between 1990 and 2017 was significantly higher than the global level. Thus, the DALY rate for oral cancer in China was characterized by rapid growth.

There are significant differences in the oral cancer data in terms of gender and age. The incidence of oral cancer increases with age. Regarding sex, the incidence rate among males is much higher than that among females. The differences in oral cancer by age and sex are consistent with findings from previous studies [26]. The reason for these differences may be that oral cancer is the result of long-term stimulation of certain risk factors, especially smoking, alcohol use and betel quid chewing [11, 27], and the proportion of men exposed to these risk factors is much higher than that of women.

The age-standardized DALY rates of oral cancer varied across provinces in China, with the greatest difference being a multiple of three between the highest and lowest provinces. The different distribution of risk factors in different provinces may be part of the reason for the variation in the burden of oral cancer among provinces [28]. High DALY rates are particularly observed in provinces with cultural practices of tobacco use, alcohol use and betel quid chewing. Surveys showed that the smoking prevalence in Yunnan, Hunan, and Guizhou provinces was relatively high compared with the national average, and there was much more economic reliance on the production of tobacco in these compared with other provinces [29, 30]. Another report showed that Sichuan is the province with the highest levels of alcohol consumption [31]. In addition, Hunan Province is recognized as one of the provinces in Chinese mainland where betel quid chewing is most prevalent [32].

This result for China is not surprising. Over the past three decades, Chinese society has experienced tremendous and rapid development. The population ageing and changes in lifestyle have had a significant impact on public health. With the popularity of vaccines and the improvement of living conditions in China, the burden of infectious diseases has declined significantly, but the burden of some cancers, including oral cancer, is steadily increasing [33].

Smoking is the most crucial risk factor for oral cancer [27]. The study by Yao et al. compared smokers with non-smokers and showed that Chinese smokers have a prominently increased risk of developing oral cancer, and the risk of oral cancer due to smoking could increase significantly with the increase in years or frequency of smoking [34]. The prevalence of oral cancer in daily smokers was three times higher than that in non-smokers. According to the 2015 tobacco report, the tobacco epidemic in China continues to be serious [35]. Smokers in China accounted for 27.7% of the total number of adults, including 52.1% of men and 2.7% of women. These different smoking habits may be the main reason for the gender and provincial disparity in the incidence of oral cancer.

Alcohol use is also an important risk factor for oral cancer [36]. Epidemiological studies have shown that drinking alcohol increases the risk of oral cancer by three-fold, which is an independent risk factor, with risk increasing with increased alcohol consumption. Compared with smoking alone, smoking combined drinking increased the risk of oral cancer by about eight-fold [37]. The 2014 statistics revealed that approximately 40.9% of oral and pharyngeal cancers worldwide were related to alcohol consumption [38]. Between 1990 and 2017, alcohol use increased significantly in some developing countries, such as Vietnam, India and China, reaching even higher levels than those found in some European regions [39]. The 2016 report showed that the per capita consumption of alcohol in China increased by 76% from the 1990 level, reaching 4.1 L in 2005, 7.1 L in 2010 and 7.2 L in 2016 [40]. Reducing alcohol consumption is very important for the prevention and control of oral cancer.

Betel quid chewing is the most common risk factor for oral cancer in China [41], and betel quid has been recognized as a first-class carcinogen by the IARC [36]. Hundreds of millions of people around the world use different types of betel quid, mainly distributed in South-eastern and Southern Asia, particularly India, Pakistan, and China [42]. Hunan Province is among the regions with the highest use of betel quid in China [9]. The raw betel nuts used in Hunan Province are imported from Hainan and Thailand, but the vast majority of Chinese betel quid products are produced and processed in Hunan Province [44]. Surveillance, clinical services and policies for betel quid and oral cancer should be provided to control and reverse the rapid growth of oral cancer related to betel quid products.

Lifestyle interventions are an effective way to prevent and control oral cancer. To encourage a healthier lifestyle and promote risk reduction of cancers, some targeted policies and regulations have been formulated and promulgated by the Chinese government, including the Healthy China 2030 Plan [45, 46], the “National Medium to Long Term Plan for Prevention and Treatment of Chronic Disease (2017–2025)” [47], national-level demonstration zones for the comprehensive prevention and control of chronic noncommunicable diseases and the Healthy Lifestyle Action (2019–2025) proposal [48]. These policies can decrease the risk of various cancers, including oral cancers, and have played a major role in some aspects of health promotion. In addition, in view of the risk factor for betel quid chewing, the Chinese government has strengthened the screening, prevention and control of this activity in key areas. In 2019, the central government announced the launch of the country’s first clinical screening programme for oral cancer in Hunan Province. The Healthy Lifestyle Action (2019–2025) also proposed strengthening oral education and oral health examinations in regions where betel quid chewing is prevalent.

There are certain limitations of this article that we need to consider [14–17, 20]. First, this study was based on the GBD study, so the limitations described in detail from the GBD 2017 study are shared by this study. Second, currently, the DSP system in China has been improved by the method of death distribution, which will likely create high deviations in disease death estimation. Third, despite the continuous improvement in cancer registration data, there were still a large number of poorly defined cancer cases that needed to be reassigned to all types of cancers, which may lead to a certain degree of deviation. In addition, revisions and changes in the coding systems over time may introduce artificial differences in disease estimates. Fourth, changes in diagnostic techniques may affect the diagnostic criteria of some diseases. Fifth, few areas in remote and poor counties were covered by disease surveillance systems, which led to a lack of reliable data on cause-specific mortality in some provinces, affecting the accuracy of our estimates. Because of the general lack of cancer registries in rural areas, as these are usually set up in urban areas, the representation of the resulting population may also be problematic. Sixth, in our study, YLLs were independent of YLDs, so the uncertainty in DALY may be undervalued. Seventh, in the GBD study, an accurate MIR was required to estimate the mortality of cancer. We further revised our estimation method of MIR for GBD 2017, but this method was still biased due to missed diagnosis cases or uncertainty deaths. Eighth, this study still lacked detailed explanations for the relationship between the burden of oral cancer and risk factors at the provincial level, thus, more in-depth studies are needed in the future.

## Conclusions

Between 1990 and 2017, the burden of oral cancer in China rapidly increased, accompanied by significant gender and age differences, which made the prevention and control of oral cancer more difficult in China. The disease burden of oral cancer and the trends in its occurrence varied greatly from province to province. These conditions deserve attention. Early screening, lifestyle intervention, oral health care, and regular oral check-ups can improve and reduce the burden of oral cancer.

## Data Availability

The datasets used during the current study are available from the GHDx website: http://ghdx.healthdata.org/data-type/disease-registry.

## Abbreviations

CDC: Chinese Center for Disease Control and Prevention
Codem: Cause of Death Ensemble modelling tool
DALY: disability-adjusted life year
DSP: Disease Surveillance Point
GATHER: Guidelines for Accurate and Transparent Health Estimates Reporting
GBD: Global Burden of Disease (study)
GHDx: Global Health Data exchange
IARC: International Agency for Research on Cancer
ICD: International Classification of Disease
MIR: mortality-to-incidence ratio
NCCR: National Central Cancer Registry
UI: uncertainty interval
WHO: World Health Organization
YLDs: years lived with disability
YLLs: years of life lost
